# Efficacy and safety of 10% HES 130/0.4 versus 10% HES 200/0.5 for plasma volume expansion in cardiac surgery patients

**DOI:** 10.1186/cc9507

**Published:** 2011-03-11

**Authors:** C Ertmer, H Van Aken, H Wulf, P Friederich, C Mahl, F Bepperling, M Westphal, W Gogarten

**Affiliations:** 1 University Hospital, Münster, Germany; 2 Phillips-University of Marburg, Germany; 3 University Hospital of Hamburg-Eppendorf, Hamburg, Germany; 4 Fresenius Kabi, Bad Homburg, Germany

## Introduction

Hydroxyethyl starch (HES) solutions are commonly used for perioperative volume replacement. Whereas older HES specimens tended to accumulate in the plasma and to cause negative effects on haemostasis, more recent products (for example, HES 130/0.4) are characterised by improved pharmacological properties. The present study was designed to compare the efficacy and safety of 10% HES 130/0.4 and 10% HES 200/0.5.

## Methods

In this *post-hoc *analysis of a prospective, randomised, double-blind, multicenter therapeutic equivalence trial, 76 patients undergoing elective on-pump cardiac surgery received perioperative volume replacement using either 10% HES 130/0.4 (*n *= 37) or 10% HES 200/0.5 (*n *= 39) up to a maximum dose of 20 ml/kg.

## Results

Equivalent volumes of investigational medications were infused until 24 hours after the first administration (1,577 vs. 1,540 ml; treatment difference 37 [-150; 223] ml; *P *< 0.0001 for equivalence). Whereas standard laboratory tests of coagulation were comparable between groups, von Willebrand factor activity on the first postoperative morning tended to be higher following treatment with 10% HES 130/0.4 as compared with 10% HES 200/0.5 (*P *= 0.025), with this difference being statistically significant in the per-protocol analysis (*P *= 0.02). Treatment groups were comparable concerning other safety parameters and the incidence of adverse drug reactions. In particular, renal function was well preserved in both groups.

## Conclusions

10% HES 130/0.4 was equally effective and safe as compared with 10% HES 200/0.5 for volume therapy in patients undergoing cardiovascular surgery. Postoperative coagulation and renal function, as measured by standard laboratory tests, were similar among groups.

